# Pre-pandemic trajectories of depressive symptomatology and their relation to depression during the COVID-19 pandemic: longitudinal study of English older people

**DOI:** 10.1192/bjo.2023.586

**Published:** 2023-10-20

**Authors:** Jingmin Zhu, Paola Zaninotto, Giorgio Di Gessa

**Affiliations:** Department of Epidemiology and Public Health, University College London, London, UK

**Keywords:** Depressive disorders, history of psychiatry, epidemiology, statistical methodology, health economics

## Abstract

**Background:**

Although the COVID-19 pandemic has affected depression, evidence of the role of pre-pandemic history of depression remains limited.

**Aims:**

We investigated how long-term trajectories of depressive symptomatology before the COVID-19 pandemic were related to depression during the pandemic, over and above the latest pre-pandemic depression status. Furthermore, we examined whether those experiencing depression closer to the pandemic were at higher risk during the pandemic.

**Method:**

Employing data from waves 4–9 of the English Longitudinal Study of Ageing (2008–2009 to 2018–2019), we used group-based trajectory modelling on 3925 English older adults aged 50+ years to identify distinctive trajectories of elevated depressive symptoms (EDS). Fully adjusted logistic models were then used to examine the associations between trajectories and depression during the COVID-19 pandemic (June–July and November–December 2020).

**Results:**

We identified four classes of pre-pandemic trajectories of EDS. About 5% were classed as ‘enduring EDS’, 8% as ‘increasing EDS’, 10% as ‘decreasing EDS’ and 77% as ‘absence of EDS’. Compared with respondents with absence of EDS, those with EDS history were more likely to have depression during the COVID-19 pandemic, particularly those with enduring or increasing EDS in the previous 10 years. Moreover, the frequency of EDS was more crucial in predicting the risks of depression during the pandemic than the timing of the latest episode.

**Conclusions:**

Trajectories of depressive symptomatology are an important risk factor for older adults’ mental health, particularly in the context of crisis. Older people with enduring or increasing EDS should receive particular attention from policy makers when provisioning post-pandemic well-being support.

Since the beginning of 2020, there have been concerns about the impact of the COVID-19 pandemic and related circumstances on mental health,^1,2^ with evidence suggesting an overall decline in mental health.^3^ As initial restrictions and stay-at-home orders were issued for at-risk individuals and older people in particular, many studies have focused on mental health sequelae of the COVID-19 pandemic among those aged 50 years and over. In addition, for this group, evidence suggests a substantial deterioration in mental health and well-being during the COVID-19 pandemic in different contexts and settings, including more prevalent self-perceived stress, loneliness or depressive symptoms.^4,5^ Abundant evidence has been provided in the literature of how older adults’ mental health was affected by disruptions and policies restricting social contact and human interaction during the pandemic. Scholars have investigated variations in mental health status during the pandemic by demographic and socioeconomic groups.^3,5,6^ An increasing number of studies have also shown how deterioration in mental health for older adults was exacerbated by, among other factors, social isolation and shielding,^6–9^ or disruptions in social or healthcare services.^10^ Although pre-pandemic mental health affected lives during the COVID-19 pandemic,^7^ important lacunae remain about the role that pre-pandemic mental health has had on the risk of mental distress during the COVID-19 pandemic. It has been suggested that older adults with prior mental disorders were more likely to report mental health problems during the COVID-19 pandemic. However, to date, most studies have either been qualitative^11^ or simply accounted for pre-pandemic mental health measured at one point in time,^12–18^ failing to take a longer view of mental health histories. Despite the importance of adopting a longer-term perspective in understanding the role of prior history of depression,^19^ to our knowledge, only a few studies have so far taken into account mental health trajectories. Pan and colleagues,^20^ using data collected between 2006 and 2016, constructed two indices for the burden of mental health disorders, accounting for the number and chronicity of mental health disorder diagnoses, respectively. Their study showed that respondents who had reported a higher number of mental health disorders in previous years, as well as those who reported them more chronically, were also significantly more likely to report mental disorders during the pandemic.^20^ Despite this significant contribution, however, these indicators were quite crude and did not take into account different patterns of mental health over time, which could capture changes in the development of mental disorders (including recovery and deteriorating) as well as individual resilience or chronic poorer mental health. The implications of their findings were also limited by the fact that the participants were recruited from communities, primary care practices and specialised mental healthcare institutions, and most of them had a mental disorder. More recently, Moreno-Agostino et al^21^ addressed some of these issues and accounted for long-term trajectories of distress across cohorts and sexes using three nationally representative British birth cohorts, i.e. the 1946 National Survey of Health and Development, the 1958 National Child Development Study (NCDS) and the 1970 British Cohort Study (BCS70). The authors concluded that pre-existing long-term distress trajectories were altered during the first year of the COVID-19 pandemic, in most cases reaching the highest average levels over the respondents’ life courses.^21^ However, although the authors examined overall trajectories of distress in each birth cohort, they did not address the issue of heterogeneity of trajectories within cohorts. Extending this work, Moulton et al^22^ examined patterns of distress within each birth cohort using NCDS and BSC70. Their study, which considered longitudinal mental health during the 30 years preceding the COVID-19 pandemic, highlighted the importance of both the severity and chronicity of psychological distress history, as both higher frequency of pre-pandemic episodes of poor mental health and poor mental health episodes occurring closer to the pandemic increased the risk of poor mental health during the pandemic.^22^ However, the sample used by Moulton and colleagues was restricted to those born in two specific years (1958 or 1970) and focused mostly on early adulthood and early- to midlife trajectories of psychological distress. As such, a research gap remains with regard to trajectories of pre-pandemic mental health occurring from middle age and throughout old age, particularly as older adults were disproportionately affected by stay-at-home policies during the COVID-19 pandemic.

Therefore, in this study, we contribute to the current knowledge on how long-term trajectories of depressive symptomatology before the COVID-19 pandemic were related to mental health status during the pandemic using nationally representative data of older adults aged 50 years and older and living in private households in England. We further investigate the role of trajectories of depressive symptomatology prior to the pandemic on depression during the pandemic, over and above the latest pre-pandemic measure of depression. Finally, we investigate the roles of both the number and the timing of depressive symptoms to shed light on whether those who experienced depressive symptoms closer to the pandemic were at a higher risk of depression during the pandemic, given the same history of depression.

## Method

### Study design and participants

Data were obtained from the English Longitudinal Study of Ageing (ELSA), an interdisciplinary ongoing cohort of older adults aged 50 years and older living in private households in England. ELSA was initiated in 2002 and followed up its participants every 2 years, using face-to-face interviews to collect a wide range of information including on health, well-being, and social and economic circumstances. The most recent pre-pandemic data are from ELSA wave 9 in 2018–2019. Soon after the outbreak of the COVID-19 pandemic, ELSA collected information on the experience of older English people during the pandemic in June–July 2020 and again in November–December 2020, via online survey or telephone (with 83% of the interviews completed online and 17% on the phone). The response rate was around 75% in each COVID-19 sub-study.

Our sample consisted of participants from at least ELSA COVID-19 sub-study 1 who had also been successfully interviewed between waves 4 (2008–2009) and 9 (2018–2019). We did not include waves 1–3 in this analysis because a large refreshment sample was added at wave 4.^23^ The final analytical sample consisted of 3925 participants present in the first ELSA COVID-19 sub-study (June–July 2020) and waves 4 to 9; of these, 3648 participants were also interviewed in the second ELSA COVID-19 sub-study (November–December 2020).

The authors assert that all procedures contributing to this work comply with the ethical standards of the relevant national and institutional committees on human experimentation and with the Helsinki Declaration of 1975, as revised in 2008. ELSA waves 4–9 were approved by the South Central – Berkshire Research Ethics Committee (17/SC/0588; 15/SC/0526), the NRES Committee South Central – Berkshire (13/SC/0532; 11/SC/0374), the Berkshire Research Ethics Committee (09/H0505/124), and the National Hospital for Neurology and Neurosurgery and Institute of Neurology Joint Research Ethics Committee (07/H0716/48). The ELSA COVID-19 sub-study was approved by the UCL Research Ethics Committee. All participants provided written informed consent.

We affirm that the manuscript is an honest, accurate and transparent account of the study being reported; that no important aspects of the study have been omitted; and that any discrepancies from the study as planned (and, if relevant, registered) have been explained.

### Main measurements of interest

#### Mental health

Mental health was measured using the eight-item short version of the Center for Epidemiologic Studies Depression (CES-D) scale, which has been validated as a reliable measure of depressive symptomatology in older adults.^24^ The scale includes eight binary (no/yes) questions that enquire about whether respondents experienced any depressive symptoms, such as feeling sad or having restless sleep, in the week before the interview. We classified respondents who reported four or more depressive symptoms on the CES-D scale as having elevated depressive symptoms, which indicates clinically significant levels of depression.^5^

#### Covariates

Based on a review of the literature, a number of individual demographic, socioeconomic and health characteristics collected at ELSA wave 4 in 2008–2009 were used as covariates.^8,25,26^ In particular, age was modelled as a categorical variable, distinguishing those aged 50–59, 60–69 and 70+ years. We also controlled for sex, marital status (single, never married; married or partnered; separated, divorced or widowed) and ethnicity (White versus non-White participants). Socioeconomic circumstances were measured by education (low: below university; high: university and above), employment status (in paid work versus not) and wealth quintiles. We calculated participants’ wealth quintile based on total net non-pension non-housing wealth. We further controlled for health, measured using a number of variables including memory, self-reported health (‘poor or fair’ versus at least good), smoking (never smoked, ex-smoker or current smoker), drinking (daily versus not), physical activity level (inactive versus at least moderately active) and self-reported quality of sleep (‘poor or fair’ versus at least good).

### Statistical analysis

#### Grouped-based trajectory modelling

First, we calculated the percentages of respondents with elevated depressive symptoms at each wave under study. Group-based trajectory modelling^27^ was then applied to identify distinctive trajectory patterns of depression using the sample of 3925 core members at waves 4–9 and at least COVID-19 sub-study 1. This method takes into account the dependency of observations and assumes a mixture of sub-populations with different individual trajectories within the target population and identifies distinctive groups within which individuals share similar developmental trajectories.^28,29^ A typological summary of longitudinal course types for depression would offer a more precise classification of individuals than that available from any single characteristic captured cross-sectionally or any crude summary measures over time.

To determine the number of trajectory groups within our sample, we fit a series of group-based trajectory models with up to six groups. Missing data were handled using full information maximum likelihood estimation. The final number of trajectory groups was decided based on comparing a range of goodness-of-fit criteria, where lower scores indicate (relatively) better fitting models (Akaike information criterion, Bayesian information criterion (BIC) and sample-size-corrected BIC).^27,30^ Moreover, we also considered the average posterior probabilities of group membership as a measure of classification quality (entropy index,^31^ with values approaching 1.0 indicating a favourable classification); group size membership (and the avoidance of too small classes that may lead to lack of reproducibility of the results, with 5% used as a cut-off^28^); the usefulness of the number of groups in terms of the similarities and/or differences in their trajectory shapes; and the interpretability of the distinctive trajectories.^27,29^

#### Multiple imputation of missing data

Missing values were detected in our outcome of depressive symptomatology measured in the COVID-19 sub-study as well as in six covariates (namely, education, wealth quintiles, self-reported health, memory, smoking and self-reported poor sleep). The percentage of missing values varied from 0.03% (in education) up to 2.3% (in wealth quintile). The multiple imputation using chained equations method was employed under the assumption of missing at random (MAR) to impute missing values in depressive symptomatology (binary variable) at COVID-19 sub-studies 1 and 2 and six covariates.^32^ To strengthen the MAR assumptions, auxiliary variables were included in the imputation model. With ten within-time iterations and ten among-time iterations, 20 data-sets were imputed. The performance of multiple imputation was assessed by comparing the distributions of the original and imputed data-sets and calculating relative efficiency.^33,34^

Regression models were then employed to analyse the 20 imputed data-sets^35^ and investigate the relationship between depressive symptomatology during the COVID-19 pandemic (in June–July or November–December 2020) and long-term trajectories of depressive symptomatology after adjusting for demographic, socioeconomic and health characteristics at baseline (i.e. wave 4, 2008–2009). Furthermore, survey weights were applied to mitigate the bias from differential non-response rates to the COVID-19 sub-studies among wave 9 (core member) respondents.

#### Robustness checks

Five robustness checks were conducted using an alternative estimation method or alternative measurement of depression history. First, longitudinal analysis was implemented, allowing for a random slope of long-term trajectory of depressive symptomatology across two COVID-19 sub-studies. Second, a continuous measure of CES-D score during the COVID-19 pandemic was used as an outcome to estimate the association between long-term trajectory of depressive symptomatology and depression during the pandemic. Third, we included the most recent pre-pandemic depression status (measured at ELSA wave 9) as an additional covariate in the models to enable us to understand the significance of the pre-pandemic trajectories over and above the last reported depression status. Fourth, the total number of reports of depressive symptomatology over waves 4 to 9 was computed as an alternative measure of mental health history then categorised into never (0), once (1), twice (2) and at least three times (3+). Its association with significant depressive symptoms during the COVID-19 pandemic was estimated. Last, focusing on respondents with at least one report of pre-pandemic depressive symptomatology, we also measured and controlled for how recently respondents had last reported elevated depressive symptoms. This was calculated as the number of months elapsed between the month of the most recent reported pre-pandemic depressive symptomatology and the month of the COVID-19 ELSA sub-study interview. Results of robustness checks can be found in the Supplementary Material available at https://doi.org/10.1192/bjo.2023.586. All the analyses were performed using Stata 18.

## Results

### Baseline characteristics of the analytical sample at ELSA wave 4

The characteristics of the respondents measured at ELSA wave 4 in 2008–2009 are shown in Table 1. Of 3925 older adults aged 50 years and above, more participants (43.5%) in our analytical sample were aged 60–69 years in 2008–2009. Slightly over half participants (56.2%) were female, and almost three-quarters (72.5%) were married or partnered in 2008–2009. Almost 84% of our participants reported good health, and over 90% participants reported being physically active. Quality of sleep was rated as poor or fair by 21.5% of ELSA participants.

**Table 1:** Baseline characteristics of the analytical sample measured in 2008–2009 (*N* = 3925)^a^

	Percentage
Age group, years	
50–59	38.8
60–69	43.5
70+	17.7
Sex	
Male	43.8
Female	56.2
Ethnicity	
White	97.4
Non-White	2.6
Marital status	
Single, never married	6.0
Married or partnered	72.5
Separated, divorced or widowed	21.5
Education^b^	
Low (below bachelor)	77.9
High (bachelor and above)	22.1
Employment status	
Not in paid work	52.2
In paid work	47.8
Wealth quintile^b^	
Lowest	20.0
Second	20.0
Third	20.0
Fourth	20.0
Highest	20.0
Self-reported health^b^	
At least good	83.6
Poor or fair	16.4
Memory test^b^	
Mean (s.d.)	11.6 (3.0)
Smoking status^b^	
Never smoked	43.4
Ex-smoker	45.9
Current smoker	10.7
Daily drinking	
No	85.3
Yes	14.7
Physical activity level: moderate or vigorous	91.9
inactive	8.2
Quality of sleep^b^	
At least good	78.5
Poor or fair	21.5

a. Source: English Longitudinal Study of Ageing wave 4 (2008–2009) (*N* = 3925). Sample restricted to participants in waves 4–9 who were successfully interviewed in the first COVID-19 sub-study.

b. Variables with missing values at baseline; percentages are based on 20 imputed data-sets.

### Distinctive long-term trajectories of depressive symptomatology

**Fig. 1:**
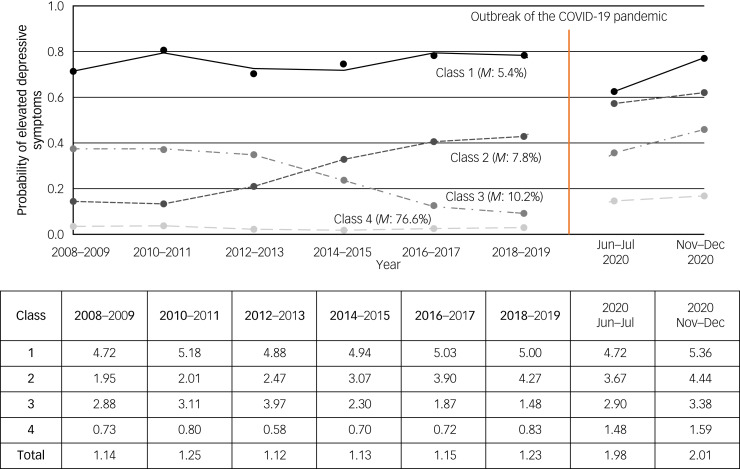
Long-term trajectories of depressive symptomatology from 2008–2009 to 2018–2019, prevalence of elevated depressive symptoms during the COVID-19 pandemic by trajectory and average values of CES-D scale scores from 2008–2009 to 2020. Source: ELSA waves 4–9 and COVID-19 sub-studies 1–2 (*N* = 3925). Class 1: enduring elevated depressive symptoms; class 2: increasing elevated depressive symptoms; class 3: decreasing elevated depressive symptoms; class 4: absence of elevated depressive symptoms. *M*, class membership. The average scores on the CES-D scale in each class are reported from 2008–2009 to 2020 in the table.

Based on the model fit indices reported in Table 2, we classified respondents’ trajectories of depressive symptomatology into four homogenous subgroups, as these provided the best model fit (as defined above). For ease of interpretation, Fig. 1 shows the prevalence of elevated depressive symptoms at each of the waves under study for each of the four latent classes. In particular, respondents in class 1 showed high probabilities of elevated depressive symptoms throughout, labelled as having ‘enduring elevated depressive symptoms’ (class 1, class membership: 5.4%). In a similar way, the other three classes were labelled as ‘increasing elevated depressive symptoms’ (class 2, 7.8%), ‘decreasing elevated depressive symptoms’ (class 3, 10.2%) and ‘absence of elevated depressive symptoms’ (class 4, 76.6%). The entropy statistic was 0.741, indicating clear delineation of classes.^31^ The average values on the CES-D scale for each class at six pre-pandemic waves (over 2008–2009 to 2018–2019) are also reported in the table in Fig. 1. The average CES-D scale values for respondents in class 1 ranged from 4.72 to 5.18 prior to the pandemic, compared with those of respondents in class 1 who reported on average fewer than one symptom throughout the years considered. Characteristics of the study sample for each class are presented in Supplementary Table 1. Overall, compared with those without depressive symptomatology, participants with ‘enduring elevated depressive symptoms’ were more likely to be female; separated, divorced or widowed; not in paid work; and in the lowest wealth quintile; and to report generally poorer health and health behaviours.

**Table 2:** Comparison of goodness-of-fit criteria for group-based trajectory modelling models of longitudinal depression^a^

No. of classes	AIC	BIC	c-BIC	Entropy	Class membership
1	−7664.04	−7684.18	−7679.72	1	100%
2	−6398.45	−6442.76	−6432.95	0.876	86.4%; 13.6%
3	−6346.35	−6414.83	−6399.67	0.664	30.4%; 63.3%; 6.3%
**4**	**−6329**.**10**	**−6421**.**74**	**−6401**.**24**	**0**.**741**	**7.8%**; **76.6%**; **10.2%**; **5.4%**
5	−6330.52	−6447.33	−6421.48	0.726	6.7%; 72.7%; 4.5%; 10.9%; 5.2%
6	−6331.03	−6472.01	−6440.81	0.708	3.3%; 68.6%; 13.3%; 6.7%; 6.6%; 1.5%

AIC, Akaike information criterion; BIC, Bayesian information criterion; c-BIC, sample-size-corrected BIC. Best-fitting solutions with interpretable distinctive trajectories are shown in bold.

a. Source: ELSA waves 4–9 (*N* = 3925).

### Mental health during the COVID-19 pandemic

Overall, 21.2% of participants reported depression in COVID-19 sub-study 1 in June–July 2020, and 23.0% in COVID-19 sub-study 2 in November–December 2020. The average values on the CES-D scale were 1.98 in June–July 2020 and 2.01 in November–December 2020, notably higher than the value of 1.23 in 2018–2019 before the pandemic (Fig. 1). However, there were substantial variations by long-term trajectory of depressive symptomatology, with clear dose–response relationships in both COVID-19 sub-studies. For instance, in June–July 2020, 62% of the respondents in class 1 (enduring elevated depressive symptoms) reported elevated depressive symptoms, with an average CES-D scale value of 4.72 compared with 14% for those in class 4 (absence of elevated depressive symptoms), who reported on average 1.48 CES-D symptoms. However, whereas the average number of depressive symptoms reported during the COVID-19 pandemic remained fairly stable for classes 1 (enduring elevated depressive symptoms) and 2 (increasing elevated depressive symptoms) compared with the pre-pandemic wave (2018–2019), classes 3 (decreasing elevated depressive symptoms) and 4 (absence of elevated depressive symptoms) experienced more substantial increases in depressive symptomatology.

### Regression results

**Fig. 2:**
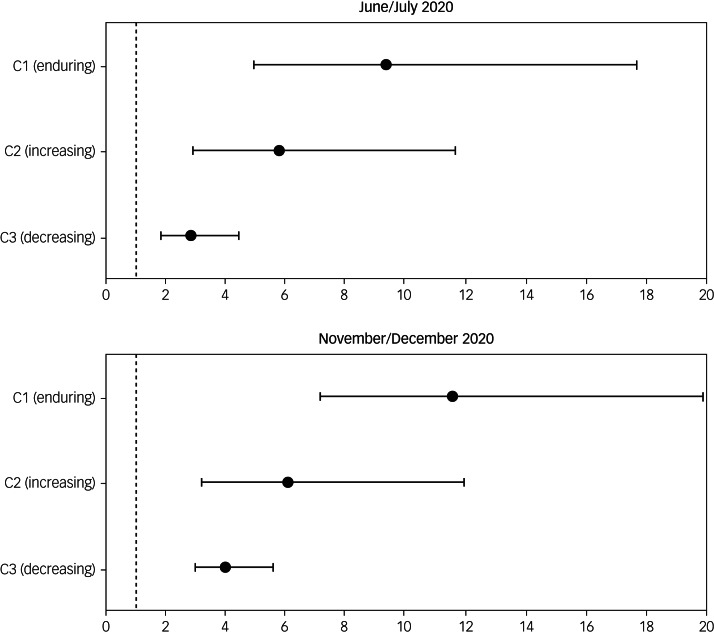
Fully adjusted odds ratios for latent classes of long-term trajectories of depressive symptomatology. Source: ELSA waves 4–9 and COVID-19 sub-studies 1–2 (*N* = 3925). Class 1: enduring elevated depressive symptoms; class 2: increasing elevated depressive symptoms; class 3: decreasing elevated depressive symptoms; class 4 (reference group): absence of elevated depressive symptoms. Outcome variables are depressive symptomatology in June–July 2020 and November–December 2020, respectively. Analyses are fully adjusted for baseline age group, sex, ethnicity, education, marital status, employment status, wealth quintile, memory status, self-reported health, smoking status, daily drinking, physical activity level and sleep quality. Survey weights are applied.

As shown in Fig. 2 (with full results presented in Supplementary Table 2), compared with older adults with ‘absence of elevated depressive symptoms’ before the pandemic, the fully adjusted odds ratios for depression in those with ‘enduring elevated depressive symptoms’ were 9.3 times (95% CI: 4.92–17.66) higher in June–July 2020 and 11.5 times (95% CI: 7.29–18.19) higher in November–December 2020. Older adults with ‘increasing elevated depressive symptoms’ before the pandemic also had 5.8 times (95% CI: 2.88–11.64) higher odds of experiencing depressive symptoms in June–July 2020 and 6.1 times (95% CI: 4.00–9.18) higher odds in November–December 2020. Furthermore, the fully adjusted analysis showed that even older adults with ‘decreasing elevated depressive symptoms’ were more likely to be depressed than those with ‘absence of elevated depressive symptoms’ (odds ratio = 2.8, 95% CI: 1.82–4.42 in June–July 2020; and odds ratio = 4.0, 95% CI: 2.93–5.45 in November–December 2020).

### Results from robustness checks

Results obtained from the longitudinal analysis and from a cross-sectional analysis which considered CES-D score as a continuous outcome were broadly consistent on the association between long-term trajectories of depressive symptomatology and depression during the pandemic (Supplementary Tables 3–4). For instance, older adults with ‘enduring elevated depressive symptoms’ tended to experience approximately three more (*b* = 2.75, 95% CI: 2.19–3.32 in June–July 2020; and *b* = 3.07, 95% CI: 2.64–3.50 in November–December 2020) depressive symptoms during the pandemic than those with ‘absence of elevated depressive symptoms’. The pre-pandemic trajectory of ‘increasing elevated depressive symptoms’ was associated with at least two more (*b* = 2.00, 95% CI: 1.48–2.51 in June–July 2020; and *b* = 2.38, 95% CI: 1.97–2.80 in November–December 2020) depressive symptoms during the pandemic. The significance of pre-pandemic trajectories was not attenuated by the inclusion of the most recent depression status before the pandemic (Supplementary Table 5). Compared with older adults without depressive symptomatology over waves 4–9 before the pandemic, those reporting depressive symptomatology on one occasion had 2.5 times (95% CI 1.69–3.60) higher risk of depression in June–July 2020, and 2.6 times higher risk (95% CI 2.01–3.35) in November–December 2020 (Supplementary Table 6). Those who reported depressive symptomatology on two occasions before the pandemic had 4.7 times (95% CI 3.06–7.23) higher odds of depression in June–July 2020, and 5.2 times (95% CI 3.64–7.41) higher odds in November–December 2020. Older adults experiencing pre-pandemic depressive symptomatology at least three times had a 9.6 times (95% CI 5.29–17.31) higher risk of depression in June–July 2020, and a 13.6 times (95% CI 9.45–19.67) higher risk in November–December 2020. Finally, when we further accounted for the timing of the most recent incidence of depression (among those who had reported it at least once pre-pandemic), we found a negligible association between how recently respondents reported pre-pandemic depression and depressive symptomatology during the pandemic (Supplementary Table 7).

## Discussion

In this study, we examined the associations between pre-pandemic trajectories of depressive symptomatology occurring during middle and old age and depression during the COVID-19 pandemic. Overall, we found a clear dose–response relationship, with older people with enduring elevated depressive symptoms in the 10 years before the pandemic considerably more likely than those with an absence of depression history to also report depression during the first year of the pandemic. This association remained when the latest pre-pandemic measure of depression was taken into account, suggesting the influence of longer-term trajectories of depressive symptomatology over and above the latest depression status and therefore adding to the importance of measuring, where possible, trajectories of depression rather than depression at one point in time.

This study extends existing evidence that pre-pandemic poor mental health aggravated mental distress during the pandemic in older adults, as found in numerous studies across the world,^13,35^ by revealing the significance of considering a long-term approach when taking pre-COVID-19 mental health into account. Although our findings support the results of many scholars who have stressed the importance of taking pre-pandemic mental health into account,^12,17,27^ we further highlight the importance of taking into account the heterogeneity of this group, and their mental health long-term histories in particular. A higher risk of depression in November–December 2020 was in line with the finding of more prevalent depressive symptoms in November–December 2020 than June–July 2020 in ELSA participants reported by Zaninotto et al in 2022.^5^ This deterioration in mental well-being could be attributed to the unexpected second national lockdown in the UK, worries about the pandemic, uncertainty about the future or more severe unemployment towards the end of 2020 than during the first national lockdown.^12^

In our study, older adults with enduring elevated depressive symptoms (as well as those who reported depressive symptomatology prior to the pandemic at least in three of the six waves under study) were much more vulnerable to mental distress during the pandemic, broadly consistent with the findings of Pan et al (2021) in Dutch adults^20^ and those by Moulton and colleagues (2023) in British adults.^21^ If anything, our study extends current knowledge on the significance of mental health trajectories by also examining trajectories of pre-pandemic depressive symptomatology occurring during middle and old age. This is particularly important as older adults were disproportionately affected by stay-at-home policies enacted during the COVID-19 pandemic. In addition, in our study, we found that the ‘severity’ of pre-pandemic depression history (including the frequency of reported episodes of depression) was more important in predicting the risk of depression during the pandemic than the timing of the last incidence of depression.

Our contribution, however, should be considered in light of some limitations. First, ELSA suffers from non-random cumulative attrition, an unavoidable problem in longitudinal studies and particularly important when studying health profiles, which was only partially corrected by using weights in the analysis. It is therefore likely that the sample analysed had an overall healthier mental health profile, as those who were depressed prior to the pandemic might have dropped out of the study or died. As a result, the prevalence of those with increasing or enduring depression profiles might have been underestimated. Second, like many studies during the COVID-19 pandemic, ELSA had to change its mode of data collection. Although questions on the CES-D scale were the same as those asked pre-pandemic, it cannot be ruled out that the change in the mode of fieldwork in the COVID-19 sub-study might have affected the prevalence of depression, given a greater willingness to disclose sensitive information (e.g. mental health status) in self-administered surveys than in face-to-face interviews.^36–38^ However, given the unprecedently high response rate, we speculate that this bias would have been minimal. Third, in this study, although we controlled for baseline age, we did not investigate whether trajectories of depressive symptomatology (and their relationships with depression during the COVID-19 pandemic) differed across different age groups. Although Moulton and colleagues^22^ found very similar trajectories and memberships across the two birth cohorts that they investigated, future studies could investigate this issue further. Last, our data were derived from observational longitudinal studies and there may be bias due to unmeasured confounding; also, our results can only be interpreted as associations, rather than causality.

Our findings have multiple implications. An unneglectable proportion of the English older population experienced persistent or increasing depressive symptoms over the course of 2008–2009 to 2018–2019, which was extremely detrimental to their mental health status. In the context of crisis, older adults who were recovering from mental illnesses can also suffer from depressive symptoms. Thus, constant interventions are required to reduce the risk of mental distress in these vulnerable older adults with profiles of depressive symptoms. In addition, more attention should be paid to long-term mental health history, particularly in those older people who show increasing or persistent poor mental health over time.

In conclusion, this study illustrates the variety in long-term trajectories of depressive symptomatology in English older adults and how these were related to depression during the COVID-19 pandemic. In our study, about one in five people experienced at least one episode of clinically significant levels of depression between 2008–2009 and 2018–2019, which placed them at higher risk of also reporting depression during the pandemic in 2020. Public authorities and healthcare professionals should therefore pay more attention to addressing in particular the mental health and wider needs of those individuals with a history of depressive symptoms, not only when emerging from the COVID-19 pandemic.

## Supporting information

Zhu et al. supplementary material Zhu et al. supplementary material

## Data Availability

The data used in this study are available from the UK Data Service with access codes SN 8688 and 5050 (https://beta.ukdataservice.ac.uk/datacatalogue/studies/study?id=5050 and https://beta.ukdataservice.ac.uk/datacatalogue/studies/study?id=8688).
