# Supplementary material for: Pre-pandemic trajectories of depressive symptomatology and their relation to depression during the COVID-19 pandemic: longitudinal study of English older people
Source: BJPsych Open. 2023 Oct 20;9(6):e195. doi: 10.1192/bjo.2023.586 (PMC10594224; doi:10.1192/bjo.2023.586)
Supplement: Zhu et al. supplementary material [file S2056472423005860sup001.docx]

**Supplementary material**

**Table S1 Baseline characteristics in 2008/9 of each class of long-term trajectories of depressive symptomatology**

|  | Class 1, %  N=202 | Class 2, %  N=155 | Class 3, %  N=294 | Class 4, %  N=3,274 | P value |
| --- | --- | --- | --- | --- | --- |
| Age group: 50-59 | 46.7 | 30.2 | 44.9 | 38.7 | 0.0179 |
| 60-69 | 28.6 | 40.1 | 38.1 | 39.7 |  |
| 70+ | 24.7 | 29.8 | 17.0 | 21.6 |  |
| Sex: Male | 29.6 | 31.3 | 30.6 | 49.7 | 0.0000 |
| Female | 70.4 | 68.7 | 69.4 | 50.3 |  |
| Ethnicity: White | 90.3 | 97.8 | 96.0 | 97.0 | 0.0091 |
| Non-white | 9.7 | 2.2 | 4.0 | 3.0 |  |
| Marital status: Single, never married | 7.7 | 3.3 | 7.5 | 5.4 | 0.0000 |
| Married or partnered | 51.8 | 62.8 | 64.0 | 74.4 |  |
| Separated, divorced or widowed | 40.5 | 33.9 | 28.5 | 20.2 |  |
| Education*: Low (Below Bachelor) | 91.3 | 92.6 | 88.1 | 81.4 | 0.0000 |
| High (Bachelor and above) | 8.7 | 7.4 | 11.9 | 18.6 |  |
| Paid employment: Not in paid work | 72.5 | 66.6 | 60.6 | 51.1 | 0.0000 |
| In paid work | 27.5 | 33.4 | 39.4 | 48.9 |  |
| Wealth quintile*: Lowest | 51.8 | 33.3 | 35.2 | 22.0 | 0.0000 |
| 2nd | 20.4 | 22.7 | 22.7 | 20.6 |  |
| 3rd | 15.3 | 22.5 | 18.6 | 19.3 |  |
| 4th | 6.5 | 12.0 | 12.6 | 19.6 |  |
| Highest | 6.0 | 9.5 | 10.9 | 18.5 |  |
| Self-reported health*: At least good | 33.6 | 67.3 | 65.2 | 85.5 | 0.0000 |
| Poor or Fair | 66.4 | 32.7 | 34.8 | 14.5 |  |
| Memory test*: Mean (SD) | 10.3 (3.3) | 10.9 (3.1) | 10.9 (3.1) | 11.5 (3.0) | 0.0000 |
| Smoking*: Never smoked | 36.6 | 39.9 | 39.5 | 41.9 | 0.0001 |
| Ex-smoker | 38.0 | 38.8 | 43.9 | 46.6 |  |
| Current smoker | 25.4 | 21.3 | 16.6 | 11.5 |  |
| Daily drinking: No | 89.0 | 89.7 | 91.6 | 85.4 | 0.0164 |
| Yes | 11.0 | 10.3 | 8.4 | 14.6 |  |
| Physical activity: Moderate or vigorous | 69.4 | 78.9 | 83.0 | 92.1 | 0.0000 |
| Inactive | 30.6 | 21.1 | 17.0 | 7.9 |  |
| Quality of sleep*: At least good | 33.6 | 65.4 | 58.3 | 83.0 | 0.0000 |
| Poor or Fair | 66.4 | 34.6 | 41.7 | 17.0 |  |

Source: English Longitudinal Study of Ageing (ELSA) wave 4 (2008/09) (N=3,925).

Notes: Class 1: Enduring elevated depressive symptoms; Class 2: Increasing elevated depressive symptoms; Class 3: Decreasing elevated depressive symptoms; Class 4: Absence of elevated depressive symptoms. For variables with missing values at baseline (denoted with *), percentages are based on 20 imputed datasets. Survey weights are applied. P value of chi-square test or t test is reported in the last column.

**Table S2 Estimates of association between long-term trajectories of depressive symptomatology and depression during the COVID-19 pandemic**

|  | COVID wave 1^a^  (Jun/Jul 2020) | | | COVID wave 2^b^  (Nov/Dec 2020) | | |
| --- | --- | --- | --- | --- | --- | --- |
|  | OR | Lower 95% CI | Upper 95% CI | OR | Lower 95% CI | Upper 95% CI |
| Long-term trajectory |  |  |  |  |  |  |
| Enduring EDS/C1 | 9.32 | 4.92 | 17.66 | 11.52 | 7.29 | 18.19 |
| Increasing EDS/C2 | 5.79 | 2.88 | 11.64 | 6.06 | 4.00 | 9.18 |
| Decreasing EDS/C3 | 2.83 | 1.82 | 4.42 | 3.99 | 2.93 | 5.45 |
| Absence of EDS/C4 | Ref. |  |  | Ref. |  |  |
| Age group |  |  |  |  |  |  |
| 50-59 | Ref. |  |  | Ref. |  |  |
| 60-69 | 1.19 | 0.82 | 1.73 | 0.89 | 0.70 | 1.12 |
| 70+ | 1.17 | 0.60 | 2.29 | 0.87 | 0.63 | 1.22 |
| Female | 1.39 | 0.90 | 2.15 | 1.51 | 1.22 | 1.85 |
| Non-white | 1.07 | 0.41 | 2.81 | 0.77 | 0.42 | 1.41 |
| High education | 1.86 | 1.16 | 3.00 | 0.99 | 0.77 | 1.28 |
| Marital |  |  |  |  |  |  |
| Single, never married | Ref. |  |  | Ref. |  |  |
| Married/Partnered | 0.86 | 0.50 | 1.47 | 0.77 | 0.53 | 1.12 |
| Separated/Divorced/Widowed | 0.75 | 0.41 | 1.39 | 0.73 | 0.48 | 1.09 |
| Paid employment | 0.99 | 0.60 | 1.63 | 0.93 | 0.74 | 1.18 |
| Wealth quintile |  |  |  |  |  |  |
| Lowest | Ref. |  |  | Ref. |  |  |
| 2nd | 0.80 | 0.49 | 1.29 | 0.87 | 0.65 | 1.17 |
| 3rd | 0.92 | 0.49 | 1.72 | 0.82 | 0.60 | 1.13 |
| 4th | 0.80 | 0.45 | 1.44 | 0.71 | 0.51 | 0.97 |
| Highest | 0.62 | 0.28 | 1.36 | 0.63 | 0.45 | 0.88 |
| Memory | 1.05 | 0.99 | 1.11 | 0.99 | 0.96 | 1.02 |
| Poor SRH | 1.20 | 0.63 | 2.31 | 1.36 | 1.03 | 1.78 |
| Smoking |  |  |  |  |  |  |
| Never | Ref. |  |  | Ref. |  |  |
| Ever | 1.04 | 0.65 | 1.67 | 0.93 | 0.76 | 1.14 |
| Current smoker | 1.48 | 0.78 | 2.79 | 1.13 | 0.80 | 1.59 |
| Daily drinking | 1.34 | 0.75 | 2.41 | 1.15 | 0.88 | 1.52 |
| Physically inactive | 1.48 | 0.84 | 2.63 | 1.54 | 1.09 | 2.18 |
| Poor sleep | 0.89 | 0.52 | 1.53 | 1.22 | 0.96 | 1.54 |
| Constant | 0.08 | 0.03 | 0.23 | 0.28 | 0.15 | 0.52 |

Source: ELSA waves 4-9 and COVID-19 sub-studies 1-2 (N=3,925).

Notes: Estimation based on 20 imputed datasets. Survey weights are applied. EDS: Elevated depressive symptoms. OR: Odds ratio. CI: Confidence Interval.

a. N=3,925. The relative efficiency (RE) of all the estimates is greater than 0.96, indicating excellent performance of MI in our analyses. The RE of three long-term trajectory estimates is 0.973, 0.968, and 0.976 respectively, very close to 1.

b. N=3,648. The relative efficiency (RE) of all the estimates is greater than 0.99, indicating excellent performance of MI in our analyses.

**Table S3 Longitudinal estimates of association between long-term trajectories of depressive symptomatology and time-varying depression during the COVID-19 pandemic**

|  | OR | Lower 95% CI | Upper 95% CI |
| --- | --- | --- | --- |
| Long-term trajectory |  |  |  |
| Enduring EDS/C1 | 12.56 | 8.43 | 18.72 |
| Increasing EDS/C2 | 7.38 | 4.74 | 11.47 |
| Decreasing EDS/C3 | 3.86 | 2.85 | 5.24 |
| Absence of EDS/C4 | Ref. |  |  |
| Age group |  |  |  |
| 50-59 | Ref. |  |  |
| 60-69 | 0.95 | 0.75 | 1.20 |
| 70+ | 0.89 | 0.66 | 1.22 |
| Female | 1.54 | 1.26 | 1.90 |
| Non-white | 0.69 | 0.41 | 1.15 |
| High education | 1.12 | 0.83 | 1.51 |
| Marital |  |  |  |
| Single, never married | Ref. |  |  |
| Married/Partnered | 0.79 | 0.54 | 1.16 |
| Separated/Divorced/Widowed | 0.71 | 0.46 | 1.08 |
| Paid employment | 0.98 | 0.77 | 1.24 |
| Wealth quintile |  |  |  |
| Lowest | Ref. |  |  |
| 2nd | 0.89 | 0.65 | 1.22 |
| 3rd | 0.86 | 0.63 | 1.19 |
| 4th | 0.71 | 0.50 | 1.01 |
| Highest | 0.61 | 0.42 | 0.90 |
| Memory | 1.01 | 0.97 | 1.05 |
| Poor SRH | 1.31 | 1.03 | 1.67 |
| Smoking |  |  |  |
| Never | Ref. |  |  |
| Ever | 0.96 | 0.78 | 1.18 |
| Current smoker | 1.10 | 0.82 | 1.50 |
| Daily drinking | 1.17 | 0.87 | 1.56 |
| Physically inactive | 1.55 | 1.16 | 2.06 |
| Poor sleep | 1.19 | 0.96 | 1.49 |
| Constant | 0.17 | 0.09 | 0.31 |

Source: ELSA waves 4-9 and COVID-19 sub-studies 1-2 (N=3,925).

Notes: Estimation based on 20 imputed datasets. Longitudinal weight is applied. EDS: Elevated depressive symptoms. OR: Odds ratio. CI: Confidence Interval.

**Table S4 Estimates of association between long-term trajectories of depressive symptomatology and CES-D score during the COVID-19 pandemic**

|  | COVID wave 1^a^  (Jun/Jul 2020) | | | COVID wave 2^b^  (Nov/Dec 2020) | | |
| --- | --- | --- | --- | --- | --- | --- |
|  | b | Lower 95% CI | Upper 95% CI | b | Lower 95% CI | Upper 95% CI |
| Long-term trajectory |  |  |  |  |  |  |
| Enduring EDS/C1 | 2.75 | 2.19 | 3.32 | 3.07 | 2.64 | 3.50 |
| Increasing EDS/C2 | 2.00 | 1.48 | 2.51 | 2.38 | 1.97 | 2.80 |
| Decreasing EDS/C3 | 1.15 | 0.82 | 1.48 | 1.68 | 1.32 | 2.05 |
| Absence of EDS/C4 | Ref. |  |  | Ref. |  |  |
| Age group |  |  |  |  |  |  |
| 50-59 | Ref. |  |  | Ref. |  |  |
| 60-69 | 0.07 | -0.21 | 0.36 | -0.09 | -0.27 | 0.10 |
| 70+ | 0.09 | -0.38 | 0.56 | -0.05 | -0.31 | 0.22 |
| Female | 0.29 | -0.10 | 0.68 | 0.41 | 0.25 | 0.56 |
| Non-white | -0.11 | -0.74 | 0.52 | -0.24 | -0.64 | 0.15 |
| High education | 0.36 | -0.08 | 0.81 | 0.17 | 0.00 | 0.35 |
| Marital |  |  |  |  |  |  |
| Single, never married | Ref. |  |  | Ref. |  |  |
| Married/Partnered | -0.17 | -0.60 | 0.26 | -0.06 | -0.36 | 0.24 |
| Separated/Divorced/Widowed | -0.22 | -0.75 | 0.30 | -0.09 | -0.43 | 0.25 |
| Paid employment | -0.11 | -0.44 | 0.23 | -0.18 | -0.37 | 0.01 |
| Wealth quintile |  |  |  |  |  |  |
| Lowest | Ref. |  |  | Ref. |  |  |
| 2nd | -0.21 | -0.58 | 0.15 | -0.02 | -0.27 | 0.23 |
| 3rd | -0.09 | -0.44 | 0.25 | -0.04 | -0.30 | 0.21 |
| 4th | -0.12 | -0.59 | 0.36 | -0.22 | -0.48 | 0.03 |
| Highest | -0.48 | -0.90 | -0.06 | -0.34 | -0.60 | -0.08 |
| Memory | 0.06 | 0.02 | 0.11 | 0.00 | -0.02 | 0.03 |
| Poor SRH | 0.18 | -0.23 | 0.60 | 0.47 | 0.22 | 0.72 |
| Smoking |  |  |  |  |  |  |
| Never | Ref. |  |  | Ref. |  |  |
| Ever | 0.18 | -0.13 | 0.50 | -0.01 | -0.17 | 0.14 |
| Current smoker | 0.17 | -0.24 | 0.58 | 0.24 | -0.04 | 0.53 |
| Daily drinking | 0.08 | -0.40 | 0.56 | 0.11 | -0.09 | 0.32 |
| Physically inactive | 0.37 | -0.07 | 0.80 | 0.28 | -0.04 | 0.59 |
| Poor sleep | 0.21 | -0.21 | 0.64 | 0.32 | 0.11 | 0.53 |
| Constant | 0.71 | -0.20 | 1.62 | 1.46 | 0.96 | 1.96 |

Source: ELSA waves 4-9 and COVID-19 sub-studies 1-2 (N=3,925).

Notes: Estimation based on 20 imputed datasets. Survey weights are applied. Outcome variable is the score of CES-D scale at COVID waves. EDS: Elevated depressive symptoms. CI: Confidence Interval.

**Table S5 Estimates of association between long-term trajectories of depressive symptomatology and depression during the COVID-19 pandemic controlling for depression at wave 9 in 2018/9**

|  | COVID wave 1^a^  (Jun/Jul 2020) | | | COVID wave 2^b^  (Nov/Dec 2020) | | |
| --- | --- | --- | --- | --- | --- | --- |
|  | OR | Lower 95% CI | Upper 95% CI | OR | Lower 95% CI | Upper 95% CI |
| **Model 1:**  **Logistic regression models with full adjustments** | | | | | | |
| Long-term trajectory |  |  |  |  |  |  |
| Enduring EDS/C1 | 9.32 | 4.92 | 17.66 | 11.52 | 7.29 | 18.19 |
| Increasing EDS/C2 | 5.79 | 2.88 | 11.64 | 6.06 | 4.00 | 9.18 |
| Decreasing EDS/C3 | 2.83 | 1.82 | 4.42 | 3.99 | 2.93 | 5.45 |
| Absence of EDS/C4 | Ref. |  |  | Ref. |  |  |
| **Model 2:**  **Model 1 with additional pre-pandemic depression at wave 9 (2018/9)** | | | | | | |
| Long-term trajectory |  |  |  |  |  |  |
| Enduring EDS/C1 | 7.38 | 3.57 | 15.27 | 5.67 | 3.28 | 9.80 |
| Increasing EDS/C2 | 4.69 | 2.15 | 10.21 | 3.19 | 1.93 | 5.26 |
| Decreasing EDS/C3 | 2.85 | 1.79 | 4.51 | 4.06 | 2.96 | 5.56 |
| Absence of EDS/C4 | Ref. |  |  | Ref. |  |  |
| EDS at wave 9 (2018/9) | 1.36 | 0.82 | 2.25 | 2.53 | 1.68 | 3.81 |

Source: ELSA waves 4-9 and COVID-19 sub-studies 1-2 (N=3,925).

Notes: Estimation based on 20 imputed datasets. Analyses are fully adjusted for baseline age group, sex, ethnicity, education, marital status, employment status, wealth quintile, memory status, self-reported health, smoking status, daily drinking, physical activity level, and sleep quality. Survey weights are applied. EDS: Elevated depressive symptoms. OR: Odds ratio. CI: Confidence Interval.

**Table S6 Estimates of association between total number of pre-pandemic depressive symptomatology and depression during the COVID-19 pandemic**

|  | COVID wave 1^a^  (Jun/Jul 2020) | | | COVID wave 2^b^  (Nov/Dec 2020) | | |
| --- | --- | --- | --- | --- | --- | --- |
|  | OR | Lower 95% CI | Upper 95% CI | OR | Lower 95% CI | Upper 95% CI |
| Total number of EDS |  |  |  |  |  |  |
| None | Ref. |  |  | Ref. |  |  |
| Once | 2.46 | 1.69 | 3.60 | 2.59 | 2.01 | 3.35 |
| Twice | 4.70 | 3.06 | 7.23 | 5.19 | 3.64 | 7.41 |
| At least three times | 9.57 | 5.29 | 17.31 | 13.64 | 9.45 | 19.67 |
| Age group |  |  |  |  |  |  |
| 50-59 | Ref. |  |  | Ref. |  |  |
| 60-69 | 1.13 | 0.78 | 1.64 | 0.89 | 0.70 | 1.13 |
| 70+ | 1.11 | 0.69 | 1.79 | 0.90 | 0.64 | 1.26 |
| Female | 1.26 | 0.91 | 1.76 | 1.40 | 1.13 | 1.72 |
| Non-white | 1.54 | 0.78 | 3.05 | 0.71 | 0.37 | 1.35 |
| High education | 1.51 | 0.91 | 2.52 | 0.97 | 0.74 | 1.25 |
| Marital |  |  |  |  |  |  |
| Single, never married | Ref. |  |  | Ref. |  |  |
| Married/Partnered | 0.73 | 0.43 | 1.24 | 0.75 | 0.51 | 1.10 |
| Separated/Divorced/Widowed | 0.62 | 0.33 | 1.17 | 0.66 | 0.43 | 1.00 |
| Paid employment | 1.03 | 0.71 | 1.48 | 0.94 | 0.75 | 1.20 |
| Wealth quintile |  |  |  |  |  |  |
| Lowest | Ref. |  |  | Ref. |  |  |
| 2nd | 0.90 | 0.54 | 1.50 | 0.92 | 0.68 | 1.24 |
| 3rd | 1.09 | 0.68 | 1.76 | 0.88 | 0.64 | 1.21 |
| 4th | 0.84 | 0.45 | 1.57 | 0.74 | 0.53 | 1.02 |
| Highest | 0.73 | 0.40 | 1.33 | 0.68 | 0.48 | 0.96 |
| Memory | 1.07 | 1.00 | 1.14 | 0.99 | 0.96 | 1.03 |
| Poor SRH | 1.24 | 0.81 | 1.91 | 1.27 | 0.96 | 1.67 |
| Smoking |  |  |  |  |  |  |
| Never | Ref. |  |  | Ref. |  |  |
| Ever | 1.07 | 0.73 | 1.57 | 0.89 | 0.72 | 1.10 |
| Current smoker | 1.28 | 0.73 | 2.22 | 1.09 | 0.77 | 1.54 |
| Daily drinking | 1.29 | 0.83 | 1.99 | 1.15 | 0.86 | 1.53 |
| Physically inactive | 1.15 | 0.62 | 2.11 | 1.51 | 1.07 | 2.13 |
| Poor sleep | 0.80 | 0.52 | 1.24 | 1.09 | 0.85 | 1.39 |
| Constant | 0.07 | 0.03 | 0.17 | 0.25 | 0.13 | 0.47 |

Source: ELSA waves 4-9 and COVID-19 sub-studies 1-2 (N=3,925).

Notes: Estimation based on 20 imputed datasets. Survey weights are applied. EDS: Elevated depressive symptoms. OR: Odds ratio. CI: Confidence Interval.

**Table S7 Estimates of association between total number of pre-pandemic depressive symptomatology, timing of the latest episode, and depression during the COVID-19 pandemic**

|  | COVID wave 1^a^  (Jun/Jul 2020) | | | COVID wave 2^b^  (Nov/Dec 2020) | | |
| --- | --- | --- | --- | --- | --- | --- |
|  | OR | Lower 95% CI | Upper 95% CI | OR | Lower 95% CI | Upper 95% CI |
| Total number of D.S. |  |  |  |  |  |  |
| Once | Ref. |  |  | Ref. |  |  |
| Twice | 3.00 | 1.15 | 7.83 | 1.20 | 0.52 | 2.77 |
| At least three times | 4.01 | 1.54 | 10.43 | 6.12 | 2.72 | 13.79 |
| Duration in months | 0.99 | 0.99 | 1.00 | 0.99 | 0.99 | 1.00 |
| Total number*Duration |  |  |  |  |  |  |
| Once | Ref. |  |  | Ref. |  |  |
| Twice | 0.99 | 0.98 | 1.00 | 1.01 | 0.99 | 1.02 |
| At least three times | 0.99 | 0.97 | 1.01 | 0.99 | 0.97 | 1.00 |
| Age group |  |  |  |  |  |  |
| 50-59 | Ref. |  |  | Ref. |  |  |
| 60-69 | 1.01 | 0.59 | 1.71 | 0.94 | 0.65 | 1.36 |
| 70+ | 1.02 | 0.50 | 2.05 | 0.91 | 0.54 | 1.53 |
| Female | 1.23 | 0.81 | 1.88 | 1.09 | 0.77 | 1.55 |
| Non-white | 1.66 | 0.57 | 4.85 | 0.37 | 0.16 | 0.84 |
| High education | 1.20 | 0.63 | 2.30 | 0.99 | 0.64 | 1.53 |
| Marital |  |  |  |  |  |  |
| Single, never married | Ref. |  |  | Ref. |  |  |
| Married/Partnered | 0.54 | 0.27 | 1.07 | 0.80 | 0.43 | 1.52 |
| Separated/Divorced/Widowed | 0.50 | 0.24 | 1.07 | 0.62 | 0.32 | 1.23 |
| Paid employment | 0.91 | 0.56 | 1.49 | 0.92 | 0.63 | 1.33 |
| Wealth quintile |  |  |  |  |  |  |
| Lowest | Ref. |  |  | Ref. |  |  |
| 2nd | 0.93 | 0.51 | 1.69 | 0.91 | 0.59 | 1.41 |
| 3rd | 1.17 | 0.60 | 2.27 | 0.94 | 0.59 | 1.51 |
| 4th | 1.02 | 0.47 | 2.21 | 0.89 | 0.53 | 1.50 |
| Highest | 0.87 | 0.42 | 1.78 | 0.87 | 0.51 | 1.49 |
| Memory | 1.07 | 0.99 | 1.16 | 0.95 | 0.90 | 1.01 |
| Poor SRH | 1.00 | 0.60 | 1.66 | 1.25 | 0.87 | 1.81 |
| Smoking |  |  |  |  |  |  |
| Never | Ref. |  |  | Ref. |  |  |
| Ever | 0.98 | 0.63 | 1.51 | 0.85 | 0.61 | 1.19 |
| Current smoker | 1.03 | 0.52 | 2.04 | 0.96 | 0.58 | 1.60 |
| Daily drinking | 0.96 | 0.52 | 1.78 | 1.02 | 0.62 | 1.67 |
| Physically inactive | 1.16 | 0.59 | 2.29 | 1.50 | 0.95 | 2.37 |
| Poor sleep | 0.83 | 0.50 | 1.37 | 1.00 | 0.72 | 1.40 |
| Constant | 0.42 | 0.09 | 1.88 | 2.07 | 0.68 | 6.34 |

Source: ELSA waves 4-9 and COVID-19 sub-studies 1-2 (N=1,139).

Notes: Estimation based on 20 imputed datasets. Survey weights are applied. EDS: Elevated depressive symptoms. OR: Odds ratio. CI: Confidence Interval.
